# Treat the Patient as a Whole, Not Just the Laboratory Findings: A Case of Contaminated Blood Cultures With Subsequent Iatrogenic Complications

**DOI:** 10.7759/cureus.72645

**Published:** 2024-10-29

**Authors:** Christ Ordookhanian, Ryan F Amidon, Max Slosarski, Paul Kaloostian

**Affiliations:** 1 Internal Medicine, University of California, Riverside School of Medicine, Riverside, USA; 2 Medical School, Medical College of Wisconsin, Milwaukee, USA; 3 Medical School, Idaho College of Osteopathic Medicine, Meridian, USA; 4 Neurological Surgery, Paul Kaloostian M.D. Inc., Pasadena, USA

**Keywords:** acute kidney injury, antibiotic administration in high risk for sepsis, antibiotic side effects, blood culture contamination, coagulase-negative staphylococci (cons), coagulase-negative staphylococcus epidermidis, iatrogenic complication, sepsis prophylaxis, sepsis risk factors, staphylococcus epidermidis bacteremia

## Abstract

Sepsis is a serious condition involving life-threatening infection-driven immune response and organ dysfunction. In the emergency department (ED), patients at high risk for sepsis or those with suspected sepsis are managed with a standardized protocol which includes empiric broad-spectrum antibiotic therapy pending blood culture results and susceptibilities, if applicable. While the benefits generally outweigh the risks, this approach may lead to complications, including allergic reactions, gastrointestinal irritation, acute kidney injury, and even financial toxicity, particularly in cases of contaminated blood cultures. We describe a case of a 68-year-old woman who presented to the ED with clinical signs of infection, and an initial workup significant for leukocytosis without a clear source of infection. Blood cultures were obtained and the patient was discharged with azithromycin. She was recommended to follow up with her primary care provider. However, the blood cultures grew coagulase-negative, gram-positive cocci and the patient was asked to return to the ED for admission. She was treated with intravenous vancomycin and subsequently developed acute kidney injury (AKI). Further investigation revealed that the blood cultures were contaminated with *Staphylococcus epidermidis *due to improper blood sampling techniques. This case highlights the risks associated with empiric antibiotic treatment of patients with suspected bacteremia, the implications associated with improper blood culture collection technique leading to false positive results, and the importance of interpreting a laboratory result within the context of the patient’s clinical status rather than relying solely on Systemic Inflammatory Response Syndrome (SIRS) criteria.

## Introduction

Sepsis refers to significant immune system activity and organ dysfunction associated with infection. Septic shock is defined as sepsis with vasopressor therapy needed for mean arterial pressure (MAP) to be kept above 65 mmHg and a blood lactate level of >2 mmol/L following initial fluid administration [1]. The most common etiologies of sepsis include *Staphylococcus aureus, Streptococcus pneumoniae, Escherichia coli, Klebsiella*, and *Pseudomonas* [2]. Most sepsis-associated infections involve the respiratory system (43%), but 14% are of unknown origin [2]. There are an estimated 50 million annual cases of sepsis globally, resulting in about 20% of all deaths, though the age-adjusted incidence and mortality are decreasing [3]. Over 850,000 patients with sepsis visit the emergency department (ED) annually and 80% of patients with sepsis are first seen in the ED [1]. It is associated with over five million deaths annually with a hospital mortality rate of 17%, increasing to 26% in septic shock [4]. However, risk-adjusted mortality shows significant variability according to location [5].

Clinical suspicion of sepsis in the ED is followed by fluid resuscitation, vasoactive agents, oxygen, and ventilation if indicated, and blood cultures with empiric antibiotic therapy if signs of instability, such as hypotension, are present [1]. Ideally, if there is a low-to-moderate risk of sepsis, antibiotics are administered within three hours following clinical evaluation. On the other hand, if there is a moderate-to-high risk of sepsis, antibiotics are administered within one hour [2]. When the infection site is unknown, the first-line treatment is a 25-30 mg/kg loading dose followed by 20 mg/kg of vancomycin given twice daily, although piperacillin/tazobactam and daptomycin are also used based on the clinical picture [2]. Daily patient assessment should occur until blood culture results are available to guide the most appropriate treatment regimen [2]. Despite prompt management being the core component of suspected sepsis care, up to 40% of patients initially treated for sepsis in the ED are diagnosed with other similarly presenting conditions (i.e., pulmonary embolism) [1], resulting in over-treatment or delay of treatment. However, given the high mortality associated with sepsis, empirical therapy is generally favored when sepsis is suspected as the benefits often outweigh the risks.

## Case presentation

A 68-year-old woman with a medical history of hypertension, hyperlipidemia, and osteoarthritis presented to the ED with concerns of fever, reaching 101.8° F at home, and chills that were only mildly resolved with ibuprofen and acetaminophen. She had suffered from mild symptoms of cold for a week but had thought that they would resolve over time. On the night she presented in the ED, the patient stated that her body ached, and the pain was becoming unbearable, particularly in her lower extremities. The last time she had similar symptoms was over two years ago when she was diagnosed with influenza.

The patient was found to have an elevated temperature of 100.6° F, along with a normal heart rate of 78 beats per minute and blood pressure of 126/81 mmHg, and a respiratory rate of 18 breaths per minute with an oxygen saturation of 97% on room air. An electrocardiogram (EKG) was performed, demonstrating normal sinus rhythm. The chest X-ray was grossly unremarkable and not demonstrative of any acute processes. General laboratory testing revealed an elevated white blood cell count of 14.5 x 10^9^/L with elevated lymphocytes. The comprehensive metabolic panel was normal except for a reduced glomerular filtration rate (GFR) which was most likely age-related. Serum lactate and procalcitonin levels were also drawn, with reference levels of <2 mmol/L and <0.1 ng/mL, respectively. The patient’s serum lactate level was 0.5 mmol/L and procalcitonin levels were 0.07 ng/mL on the first day and <0.05 ng/mL on the next. Thus both values were within their respective reference levels. 

The patient was conservatively managed with 1,000 mg of acetaminophen and one liter of intravenous (IV) isotonic normal saline bolus followed by an additional liter infused over four hours. She was monitored for several hours. During this time, a blood culture was ordered from two separate peripheral sites and sent to the hospital clinical laboratory. The patient was subsequently discharged from the ED with a 10-day course of azithromycin and instructed to follow up with her primary care physician.

Within two days of discharge, the blood culture obtained from the patient became positive and showed the presence of coagulase-negative and gram-positive cocci. The patient was instructed to return to the ED as she was considered to be at high risk for sepsis based on the presence of bacteremia. She was admitted to the hospital and was afebrile with reassuring vitals and a white blood cell count of 11.2 x 10^9^/L which showed a downward trend (Table 1). She was started on one gram of IV vancomycin every 12 hours, and the admission team discontinued azithromycin. The patient also received an additional chest X-ray that did not show any changes from the previous one.

**Table 1:** Comprehensive metabolic panels and complete blood counts ED: emergency department

Laboratory test	Day 1 (ED)	Day 3 (Day of admission)	Day 4	Day 5	Day 6	Day 7
Sodium (mEq/L)	138	136	137	141	137	136
Potassium (mEq/L)	4.2	3.9	3.8	4.0	3.7	4.0
Chloride (mEq/L)	105	104	105	106	107	107
Carbon dioxide (mEq/L)	21	23	22	24	22	21
Blood urea nitrogen (mg/dL)	19	16	14	15	14	14
Creatinine (mg/dL)	0.83	0.89	1.40	1.24	1.03	0.87
Estimated glomerular filtration rate (mL/min/1.73 m^2^)	77	73	41	47	59	73
Glucose (mg/dL)	112	107	113	109	97	97
White blood cell count (× 10^9^/L)	14.5	11.2	10.7	10.5	9.8	8.9
Hemoglobin (g/dL)	13.6	13.8	14.1	13.6	13.3	13.8
Hematocrit (%)	40.1	40.2	40.5	40.2	40.1	40.3
Platelet count (× 10^9^/L)	363	353	357	341	347	332
Neutrophils (%)	48	63	66	69	70	69
Lymphocytes (%)	46	31	29	24	21	22

The patient remained hospitalized, receiving IV antibiotics for a total of five days, until blood cultures indicating a specific organism were obtained. Her vitals and laboratory testing continued to remain normal throughout admission, with an improving white blood cell count. However, the patient’s creatinine became elevated and she was diagnosed with an acute kidney injury (AKI) on the second day of admission. On the fourth day of hospitalization, the blood cultures grew *Staphylococcus epidermidis* and the primary medicine team received a call from the hospital's clinical laboratory indicating that the result was likely a contaminant from the skin when the cultures were obtained. Additionally, the hospital's antibiotic stewardship committee reviewed the ED records and discovered that the blood cultures had come from the IV catheter sites rather than two new peripheral venous sample sites. When these findings were communicated to the primary medicine team, vancomycin was discontinued, and the patient was discharged after receiving a thorough explanation of what had happened.

## Discussion

Empiric antibiotic therapy for patients incorrectly suspected of having sepsis exposes them to adverse effects of the medications, such as AKI. AKI is measured by serum creatinine (increase by at least 0.3 mg/dL in 48 hours or by at least 1.5 times baseline within seven days) and urine volume (decrease in urine output to less than 0.5 mL/kg/h after six hours) [2]. The antibiotics vancomycin and piperacillin/tazobactam are commonly associated with this adverse effect. About 40% of patients with sepsis and 64% with septic shock experience AKI [2]. Renal replacement therapy (RRT), such as hemodialysis, may be indicated in severe AKI, with no significant difference in mortality between early or delayed treatment in patients with sepsis [2].

Contaminated blood cultures in the ED are a significant cause of unnecessary antibiotic administration and subsequent AKI. Additionally, patients with contaminated blood cultures experience an average of two days of increased hospital stay compared to those with negative cultures [6]. Hospital costs are $12,824 per patient with a contaminated blood culture compared to $8,286 per patient with a negative blood culture [6]. One study demonstrated that in 85% of cases where coagulase-negative *Staphylococci* (CoNS) are the cause of positive blood culture findings, antibiotic treatment is unnecessary due to culture contamination [4]. CoNS-positive blood cultures are frequently identified within four hours of flagging [7]. Finally, contaminated cultures leading to hospital stay extensions further expose patients to hospital-acquired infections [4].

In one study involving 3,325 patients, vancomycin was provided to 84% of patients with contaminated bacterial growths in the ED blood cultures [6]. In another study of over 30,000 blood culture results, the blood culture contaminant (BCC) group experienced greater rates of vancomycin administration and combined length of treatment compared to the no-growth group [7]. Different studies demonstrate high variability in the length of treatment, paralleling the variability in mortality rates which is likely due to institutional and regional differences in protocols. One study reported an average of 6.5 days of vancomycin treatment for the BCC group while another reported an average of 3.5 days for the BCC group compared to 2.5 days for the no-growth group [8,9].

Despite the Clinical and Laboratory Standards Institute (CLSI) recommending a contamination rate under 3%, rates range from 2% to over 10% depending on the institution [6]. Contamination rates are higher in the ED compared to the general wards and intensive care units [6]. Contributing factors include high patient volumes, high patient acuity, staff transition, and the time-sensitive nature of tasks in the ED [10]. Surveillance and feedback systems, blood culture collection kits, and the establishment of standardized protocols have demonstrated a reduction in contamination rates [10-12].

This case highlights several key learning points. Our patient was symptomatic, likely from a viral illness, with consistently stable vital signs; however, a contaminated blood culture led to an unnecessary exposure to antibiotics resulting in an AKI. Given the substantial risks associated with contaminated blood cultures, including unnecessary antibiotic exposure and financial toxicity for the patient, it is imperative for EDs to implement strategies aimed at minimizing contamination rates. Education and training for healthcare staff on proper blood collection techniques, alongside the utilization of specialized blood culture kits, can enhance the accuracy of culture results and reduce the incidence of misdiagnosis. Additionally, incorporating routine surveillance and feedback mechanisms can help identify and address potential sources of contamination, ultimately improving patient safety and outcomes. By prioritizing these measures, clinicians can better navigate the challenges of suspected sepsis management, ensuring that patients receive appropriate and timely treatment while minimizing the risks of adverse effects from unnecessary antibiotic therapy.

## Conclusions

Sepsis remains a frequently encountered condition in the ED, particularly in elderly patients, and its management can be challenging due to variability in presentation, comorbidities, and risks associated with treatment, though the benefits of empirical treatment generally outweigh the risks. Contamination of blood cultures further complicates medical decision-making, often leading to unnecessary antibiotic use and related complications, such as AKI. Ultimately, this case underscores the importance of cautious interpretation of blood culture results and judicious use of antibiotics to avoid preventable complications, such as AKI, especially in elderly patients who may be more vulnerable to adverse effects.
